# Marine Natural Compounds with Biomedical Potential: 2nd Edition

**DOI:** 10.3390/biom14081005

**Published:** 2024-08-14

**Authors:** Elena Leychenko

**Affiliations:** G.B. Elyakov Pacific Institute of Bioorganic Chemistry, Far Eastern Branch, Russian Academy of Sciences, 159, Pr. 100 let Vladivostoku, Vladivostok 690022, Russia; leychenko@gmail.com

The potential of marine natural compounds for drug design is difficult to overestimate. Marine organisms are ready to provide scientists with a vast arsenal of compounds with unique structural and biological characteristics that can address numerous challenges in biomedicine. Thus, the novelty of the structures of marine secondary metabolites with antimicrobial activity is key to solving the problem of antibiotic resistance, while the similarity of their protein folds to those of humans seems to offer a potential avenue for addressing the issue of immunogenicity. The development of new drugs based on marine natural compounds is impossible without a thorough structural and functional analysis, activity clarification, target determination, and action mechanism establishment.

This Special Issue includes six original research papers and two reviews from experts in the field, providing readers with advances in the study of marine natural products derived from marine animals, algae, and marine fungi.

In their review, Shi and co-authors [1] provide a comprehensive summary of the chemical structure types, sources, distribution, biological activities, and biological synthesis of the compounds isolated from Acrostalagmus from 1969 to 2022. According to data from the literature, terpenoids and alkaloids represent the two main structural classes of secondary metabolites in ascomycete fungi of the genus Acrostalagmus. Other classes of metabolites, such as cyclic dipeptides, pyranones, benzene derivatives, and paulownins, are less abundant. These compounds can be used in the development of new anticancer drugs, antibiotics, and other agents due to their cytotoxic, enzyme-inhibitory, and antimicrobial activities. It is noteworthy that half of the compounds isolated from the genus Acrostalagmus are new, with more than 80% belonging to marine Acrostalagmus, highlighting the biomedical potential of marine fungi.

Five new quinazolinone alkaloids, felicarnezolines A–E, a new highly oxygenated chromene derivative, oxirapentyn M, and five previously reported related compounds were found by Belousova et al. via co-cultivation of marine-derived fungi *Aspergillus carneus* KMM 4638 and *Amphichorda* sp. KMM 4639 [2]. Felicarnezoline B demonstrated anti-hypoxic activity, effectively protecting rat cardiomyocyte H9c2 cells and human neuroblastoma SH-SY5Y cells against CoCl_2_-induced damage (a hypoxia-mimicing in vitro model). It has been suggested that the cytoprotective effect of the novel antihypoxic metabolite is associated with the increased activity of superoxide dismutase, which is one of the components of the first line of antioxidant defense in the body [3].

In the study of phytoceramides from the sponge *Monanchora clathrata*, Santalova et al. were able to analyze the total ceramides, molecular ceramide species, and their sphingoid/fatty acid components using NMR spectroscopy and mass spectrometry [4]. As a result, sixteen new and twelve known compounds were identified. Phytoceramides were found to reduce the cytotoxic effect of the previously isolated alkaloid from *M. clathrata*, crambecidin 359, and cisplatin on MDA-MB-231 and HL-60 cells, as well as the level of ROS (reactive oxygen species) induced by paraquat in neuroblastoma cells. It was noted that the cytoprotective effect of phytoceramides was only manifested after being used for the preliminary treatment (for 24 or 48 h) of the cells.

The structures of three new bibenzochromenones (phanogracilins A–C) from the crinoid *Phanogenia gracilis* were determined by Vasileva et al. through the application of various structural methods, including X-ray crystallography (for phanogracilin A) [5]. The authors noted that this is the first example of C–C-connected benzochromenone molecules with demethylated hydroxyl groups, which is not typical of crinoids. The phanogracilins A, C, and compound derived from A exhibited significant antiradical properties, which were higher than those of the positive control, trolox. Moreover, phanogracilins A and C have been shown to inhibit the growth of Gram-positive bacteria *Staphylococcus aureus* and yeast-like fungi *Candida albicans*. Additionally, they prevented the formation of biofilms by these microorganisms, which suggests their potential as antimicrobial agents.

The challenges and prospects of using sulfated galactans (SGs) from agarophytes are summarized in the review by Chumsook et al. [6]. SGs from agarophytes are known to be widely used in industry as agar. Currently, they are attracting the attention of scientists due to various biological activities, such as anti-tumor, anticoagulant, anti-inflammatory, antioxidant, anti-obesity, anti-diabetic, anti-microbial, anti-diarrhea, and gut microbiota regulation properties, which have indicated that SGs have promising potential as health-improvement agents. The collected evidence in the review has indicated that variations in biological activity depend on various structural features of SGs, including molecular mass, the composition of monosaccharides, the degree of sulfated esterification, the position of sulfated esterification, and the degree of polymerization, which are affected by geographical conditions (salinity, temperature, light), and preparation methods (pretreatment, isolation, and purification methods). The authors revealed three approaches for developing SGs into products with high economic value. They believe that the fundamental factors for obtaining various types of SGs are (1) steady aquaculture and a clear understanding of biomass; (2) knowledge of the relationship between fine structure and bioactivity; and (3) conducting clinical trials. Further exploration of SGs in accordance with the above suggestions may benefit marine resource development and contribute to the growth of the blue economy.

The work by Malyarenko and co-authors continues the theme of studying algae carbohydrates [7]. Fucoidan from the brown algae *Saccharina cichorioides* and its derivatives obtained by autohydrolysis were found to reveal a radiomodifying effect in combination with pacificusoside D, a triterpene glycoside from the starfish *Solaster pacificus*. More than twenty different combinations of fucoidan, pacificusoside D, and X-rays were tested by the authors in the model of the viability and invasion of 3D melanoma SK-MEL-2 cells to determine the most effective therapeutic scheme. It included the pre-treatment of spheroids with fucoidan, followed by X-ray irradiation, and the subsequent treatment of cells with triterpene glycoside. The molecular mechanism of the radiomodifying effect was established to be associated with the induction of intrinsic apoptosis via DNA fragmentation in melanoma cells. The authors believe that the combination of the unique biological properties of brown algae with starfish metabolites in radiochemotherapy could contribute to the development of a highly effective therapeutic approach for malignant melanoma.

Natural membrane-active antimicrobial peptides (AMPs) offer an alternative way to fight pathogenic microorganisms, which can be a potential threat to new epidemics due to the emergence of multidrug-resistant strains. Cationic AMPs selectively interact with anionic bacterial membranes, disrupting the integrity of the membrane and causing bacterial cell death. It is known that the oligomerization of AMPs increases their activity and selectivity toward bacterial membranes. An NMR structural and thermodynamic study of the dimerization of the β-hairpin membrane-active cationic AMP, capitellacin (20 a.a.), from marine polychaeta *Capitella teleta* was carried out by Mironov et al. [8]. As a result, insights into the mechanism of action of capitellacin were provided. Capitellacin was found to exchange between monomeric and dimeric micelle-bound forms in the membrane-mimicking environment of dodecylphosphocholine micelles. The authors determined the spatial structures of two forms of capitellacin and also studied the concentration and temperature dependencies of the dimerization of the peptide. Dimer stability and hemolytic activity were revealed to mainly depend on the hydrophobicity of the peptide, and the formation of dimers is determined by the association of the polar or weakly hydrophobic surfaces of the molecules. The data obtained may be relevant to the processes occurring in biological membranes, and they are necessary to better understand the membrane-disrupting activity of capitellacin and similar peptides.

A novel peptide AnmTX Sco 9a-1 (28 a.a.) with a β-hairpin fold was isolated from the swimming sea anemone *Stomphia coccinea* (Actinostolidae family) by Kalina et al. [9]. In vivo tests showed that the peptide stimulated exploratory motivation and active search behavior in mice and demonstrated an anti-anxiety effect, which was a first-time find for the sea anemones’ β-hairpin toxins. A significant analgesic effect of AnmTX Sco 9a-1 was revealed in the λ-carrageenan-induced thermal hyperalgesia model. Moreover, AnmTX Sco 9a-1 exhibited anti-inflammatory activity in a model of acute local λ-carrageenan-induced inflammation. In the last test, the peptide reduced the concentration of tumor necrosis factor-α (TNF-α) up to the level observed in the intact group.

Hopefully, this Special Issue of *Biomolecules* will be appreciated by readers, and the new compounds presented will prove useful as potential medicines. In any case, the articles in this issue contain new opportunities for future technological developments.
